# L-Theanine Extends the Lifespan of *Caenorhabditis elegans* by Reducing the End Products of Advanced Glycosylation

**DOI:** 10.3390/foods14020221

**Published:** 2025-01-13

**Authors:** Zhihang Huang, Haiming Jing, Yan Pan, Hongxia Cai, Wenjing Zhang, Jingyuan Zhu, Nan Zhang, Dan Wu, Wentao Xu, Hexiang Qiu, Huihui Bao, Guojun Li, Junyu Ning, Bo Xian, Shan Gao

**Affiliations:** 1 Beijing Key Laboratory of Diagnostic and Traceability Technologies for Food Poisoning, Beijing Center for Disease Prevention and Control, Beijing 100013, China; zhihanghuang@126.com (Z.H.); tabtab1122345@163.com (H.J.); zzwwjing@163.com (W.Z.); tyzhangnan@hotmail.com (N.Z.); gj20071@sina.com (G.L.); njy_med@hotmail.com (J.N.); 2 Laboratory of Aging Research, School of Medicine, University of Electronic Science and Technology of China, Chengdu 610056, China; yanpan@zohomail.com (Y.P.); ccaijiu@163.com (H.C.); zhujingyuanailu@163.com (J.Z.); wdwudan2024@163.com (D.W.); wentaoxu2024@163.com (W.X.); 13178034985@163.com (H.Q.); 3 NHC Key Laboratory of Food Safety Risk Assessment, Chinese Academy of Medical Science Research Unit, China National Center for Food Safety Risk Assessment, Beijing 100022, China; baohuihui@cfsa.net.cn; 4 School of Public Health, Capital Medical University, Beijing 100069, China

**Keywords:** L-theanine, *Caenorhabditis elegans*, advanced glycation end products (AGEs), lifespan extension, insulin-like signaling

## Abstract

L-theanine, a non-protein amino acid naturally occurring in tea leaves, is recognized for its antioxidant, anti-inflammatory, and neuroprotective properties. Despite its known benefits, the mechanisms by which L-theanine influences lifespan extension remain poorly understood. This study investigated the effects of L-theanine on the lifespan of *Caenorhabditis elegans* and explored the underlying mechanisms. Our findings indicate that L-theanine significantly diminishes the accumulation of advanced glycation end products (AGEs), which are biomarkers closely linked to aging and age-related diseases. Through an AGE-level analysis, we observed that L-theanine, when administered during early adulthood, notably extended the lifespan of *Caenorhabditis elegans* under both normal and high-glucose-induced stress conditions. L-theanine enhanced the lifespan under typical conditions and provided protective effects against high-glucose-induced stress. A further analysis demonstrated that L-theanine extends the lifespan of *Caenorhabditis elegans* by modulating the DAF-2/DAF-16 insulin-like signaling pathway and reducing the accumulation of advanced glycation end products (AGEs). In summary, this study identified L-theanine as a potential anti-aging intervention that extends the lifespan by reducing AGE accumulation and regulating insulin-like signaling pathways. These findings provide new insights for developing anti-aging strategies and lay the groundwork for further research on the potential benefits of L-theanine in mammals. Future studies could explore the molecular mechanisms, test L-theanine in mammalian models, and assess the long-term side effects.

## 1. Introduction

Aging is a multifaceted biological process characterized by a gradual decline in physiological function and an increased prevalence of age-related diseases such as cardiovascular diseases, neurodegenerative disorders, and cancer [1,2,3]. While cellular damage and tissue function decline are inevitable aspects of aging, modulating specific molecular mechanisms to delay this process remains a focal point of scientific research. Recently, there has been a growing interest in anti-aging interventions, including dietary adjustments, pharmacological treatments, and genetic regulation.

At the organismal level, aging impacts physiological functions and is associated with the development and progression of chronic diseases [4,5]. For instance, the incidence of cardiovascular diseases rises significantly with age, due to decreased vascular elasticity, atherosclerosis, and declining cardiac function. Neurodegenerative diseases, such as Alzheimer’s and Parkinson’s, are also more prevalent among the elderly, characterized by neuronal degeneration and death and leading to severe cognitive and motor impairments. Additionally, the incidence of cancer increases with age, which is attributed to genomic instability and a decline in the immune system [6,7].

Advanced glycation end products (AGEs) are complex molecules formed through the non-enzymatic glycation of proteins or lipids [8]. This process typically begins with reducing sugars (such as glucose) reacting with amino groups in proteins, lipids, or nucleic acids, forming unstable Schiff bases and Amadori products, which, through a series of chemical reactions, eventually form AGEs [9,10]. AGE formation is a natural process, but is significantly accelerated in hyperglycemic environments, such as in diabetic patients [11,12]. The accumulation of AGEs is associated with various aging-related pathological processes. For example, AGEs can cross-link proteins, altering their function, damaging cellular structures, and contributing to the development of atherosclerosis, a major cause of cardiovascular diseases linked to myocardial infarction and stroke.

Moreover, AGEs can activate multiple cellular signaling pathways through their receptor (RAGE, receptor for advanced glycation end products), triggering inflammatory and oxidative stress responses. Chronic inflammation and oxidative stress accelerate aging and contribute to the development of neurodegenerative diseases such as Alzheimer’s and Parkinson’s [13,14]. The role of AGEs in diabetic complications is particularly prominent [15]. In diabetic patients, chronic hyperglycemia leads to accelerated AGE accumulation, resulting in tissue hardening and dysfunction and directly causing severe complications such as diabetic neuropathy, nephropathy, and retinopathy [8]. For instance, diabetic retinopathy, resulting from AGE accumulation in retinal vessels, leads to vascular dysfunction and abnormal neovascularization, causing vision loss or blindness [16,17]. Research indicates that AGEs accumulate intracellularly and directly affect cellular functions, leading to mitochondrial dysfunction, protein misfolding, and altered gene expression, all of which play crucial roles in the aging process [18,19]. Inhibiting AGE formation or promoting AGE degradation can effectively delay aging and prevent various age-related diseases [8]. Thus, understanding the mechanisms of AGE formation and finding effective AGE inhibitors are of significant scientific and clinical importance.

In *Caenorhabditis elegans* (*C. elegans*), AGE accumulation is similarly linked to aging and a reduced lifespan. Studies have shown that AGE levels significantly increase with age in *C. elegans*, correlating with physiological decline. High-glucose diets accelerate AGE formation, shortening the lifespan [20]. For example, exposure to high-glucose environments significantly raises the AGE levels in *C. elegans*, leading to a reduced lifespan and impaired motor function. These findings suggest that AGEs play a crucial role in regulating aging in *C. elegans*. Additionally, multiple studies have demonstrated that the insulin/IGF-1 signaling pathway is crucial in regulating *C. elegans*’ lifespan, with DAF-2 (an insulin-like receptor) and DAF-16 (a downstream transcription factor) being key regulators in this pathway [21,22,23]. DAF-2 mutations significantly extend the lifespan, while DAF-16 nuclear localization and activity are critical for this process [24,25]. Studying these signaling pathways can provide deep insights into the molecular mechanisms of aging and the development of potential anti-aging interventions.

L-theanine is a natural non-protein amino acid primarily found in tea leaves, with various biological activities [26]. Research shows that L-theanine has antioxidant, anti-inflammatory, and neuroprotective effects, and can improve cognitive function and reduce stress [27,28,29]. For example, L-theanine significantly improved the memory function in aged mice and reduced the oxidative stress levels in the brain [30,31,32]. Additionally, L-theanine has been shown to delay aging-related physiological changes by inhibiting AGE formation and reducing oxidative stress [33,34,35]. Some studies even suggest that L-theanine has anti-cancer properties, inhibiting the growth of certain cancer cells [36,37]. However, despite its potential in the anti-aging field, the specific mechanisms and effects of L-theanine need further investigation.

This study systematically explored the effects of L-theanine on *C. elegans*’ lifespan and revealed its potential molecular mechanisms for the first time. L-theanine reduces AGE accumulation in *C. elegans* and significantly extends the lifespan under both normal and high-glucose-induced stress conditions. This indicates that L-theanine exhibits anti-aging effects under both regular and stress conditions. Further studies revealed that this effect depends on the regulation of the DAF-2/DAF-16 signaling pathway. Our findings provide new insights into the anti-aging mechanisms of L-theanine and theoretical support for developing new anti-aging intervention strategies. This discovery lays the foundation for further research on the potential benefits of L-theanine in mammals and is expected to play an important role in future anti-aging research and applications.

## 2. Materials and Methods

### 2.1. Experimental Subjects and Treatments

L-theanine was sourced from Aladdin (CAS: 3081-61-6) (London, UK) and dissolved in ddH_2_O to prepare a 10 mM stock solution. This stock was subsequently used to prepare NGM agar plates, incorporating the stock solution to achieve a final concentration of 10 µM L-theanine in the working medium. The following *C. elegans* strains were used in this study: N2, wild-type; CB1370, daf-2 (e1370); CF1038, daf-16 (mu86); and CL2166, dvIs19 [(pAF15) gst-4p::GFP::NLS]. These were obtained from the CGC (Caenorhabditis Genetics Center, Minneapolis, MN, USA). The worms were incubated at a constant temperature of 20 °C on nematode growth medium (NGM) plates (3 g NaCl, 2.5 g peptone and 20 g agar and 1 L ddH_2_O, with 25 mL KPO_4_ buffer (pH = 6.0), 1 mL of 1 mol CaCl_2_, 1 mL of 1 mol MgSO_4_ and 1 mL of 13 mmol cholesterol solution (prepared in ethanol) added after autoclaving). NGM plates were covered with the *E. coli* strain OP50 as a food source. To simulate high-sugar conditions, 2% glucose was added to the NGM medium and OP50. The glucose was sourced from Amresco (VWR) (Solon, OH, USA). A 2% glucose concentration was prepared by adding 4 g of glucose to 200 mL of NGM medium. This ensured that the nematodes formed more AGEs when consuming the bacteria, a method consistent with the approach described in the WormCNN study [38]. The experimental setup involved dividing the worms into four distinct treatment groups to evaluate different conditions: the control group, with the standard NGM medium; the L-theanine group, with the NGM medium supplemented with 10 μM L-theanine to assess its effects; the high-glucose group, with the NGM medium supplemented with 2% glucose to study glucose’s impact; and the high-glucose + L-theanine group, with both 2% glucose and 10 μM L-theanine to explore the combined effects of these treatments. This approach allowed us to comprehensively assess the influence of L-theanine and high glucose, both individually and in combination, on the physiology and lifespan of the worms.

### 2.2. Longevity and Phenotype Measurement

Before the longevity experiment, a nematode synchronization experiment was needed. A large number of adult nematodes were washed off with S-basal and placed in a 15 mL centrifuge tube. After the nematodes settled at the bottom of the tube, the supernatant was completely aspirated. Then, 500 μL of 10% NaClO, 500 μL of 5 M NaOH, and 9 mL of ddH_2_O were added, and the nematodes were lysed for 10 min. After centrifugation and precipitation, the supernatant was aspirated, and 5 mL of S-basal was added to complete the synchronization experiment. The lifespan experiment was performed when the nematodes grew into the L1 stage the next day. The lifespan experiments were conducted at 20 °C, starting from the young adult stage, with the worms being transferred to NGM plates containing the various treatments. The survival of the worms was monitored daily until all had died. A worm was considered dead if it did not respond to gentle prodding and showed no signs of fluid extrusion. The data were analyzed statistically using the Kaplan–Meier method, with survival curves generated via the GraphPad Prism software (version 9.0.0). The log-rank test was used for a comparative analysis. The phenotypic measurements included assessments of body bending, head swinging, and pharyngeal pumping rates, providing insights into the motor function and overall health. On the fifth day, 10 nematodes from each treatment group were randomly selected for a detailed phenotypic analysis. This analysis aimed to identify any treatment-induced alterations in behavioral and physiological traits, contributing to a comprehensive understanding of the effects of L-theanine and high glucose on *C. elegans*. All the procedures were performed in triplicate to ensure the reproducibility and statistical reliability of the results.

### 2.3. AGE Detection by Fluorescence and ELISA

To detect the accumulation of AGEs in the worms [39], we prepared a 2% glucose gel and heated it to ensure complete dissolution. The hot gel was then deposited onto a slide and covered with a coverslip to form gel slides. After allowing the gel to cool and solidify, 1 μL of 16 mM levamisole hydrochloride was added to the gel. Ten worms were then placed in the gel, and once the liquid dried, the worms were aligned neatly on the surface. Using an inverted microscope equipped with the ZEN software (version 3.9.3), we captured DAPI, bright field, and merged images to assess the AGE accumulation. To ensure consistency, the exposure intensity was standardized across all the treatment groups. Post-imaging, the fluorescence intensity was quantified with the ZEN software. For the detection of AGEs using ELISA, the control and experimental groups were treated from day 0 to day 5. Post-treatment, an equal quantity of nematodes from each group was collected and subjected to ultrasonic disruption to release their cellular contents, and the supernatant was subsequently collected. The ELISA was then performed according to the instructions provided with the ELISA kit (MEIMIAN, Changzhou, China). Sample and standard substances were added to a 96-well plate. Detection antibodies and enzyme conjugates were added to the wells, followed by incubation and washing steps to remove unbound substances. A substrate solution was added to develop color, and the reaction was stopped after a specific time. The optical density (OD) at 450 nm was measured using a microplate reader. The concentration of AGEs in each sample was calculated using a standard curve derived from the known concentrations of the standards. Differences in the AGE levels among the groups were analyzed using a one-way ANOVA. The GraphPad Prism software was utilized to visualize and analyze the fluorescence and ELISA data collected.

### 2.4. Quantification of gst-4::GFP Expression

To assess the expression of gst-4::GFP, the nematodes were treated on day 0 in the following groups: control, 10 µM L-theanine alone, 2% high glucose, and 2% high glucose combined with 10 µM L-theanine. On day 5, fluorescence imaging was performed to quantify the gst-4::GFP levels. The preparation and imaging procedures were consistent with those used for the AGE measurement, ensuring the comparability and reliability of the results.

### 2.5. Statistical Analysis

The experimental data are presented as the mean ± standard error of the mean (mean ± SEM) to provide a clear representation of the variability and central tendency. To determine the statistical significance between the different experimental groups, a one-way analysis of variance (ANOVA) was employed. This method allows for the comparison of means across multiple groups simultaneously to identify any significant differences. Following the ANOVA, Tukey’s multiple comparison test was applied to perform pairwise comparisons between each group. This post hoc test helps to pinpoint which specific groups differ significantly from each other, providing a more detailed analysis of the data. A *p*-value of less than 0.05 was considered statistically significant, indicating a less-than-5% probability that the observed differences were due to random chance. All the statistical analyses were conducted using the GraphPad Prism 9.0 software, which provides robust tools for data visualization and interpretation.

## 3. Results

### 3.1. L-Theanine Prolongs Lifespan, Both With and Without Glucose Addition

**Figure 1:**
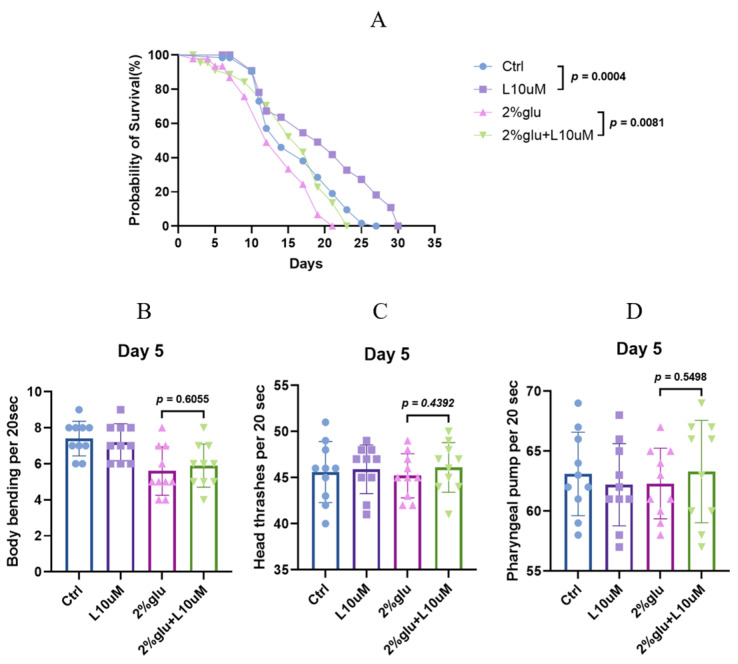
Effects of L-theanine (L) on lifespan phenotypes of *C. elegans*. (**A**) Control (Ctrl) group and 10 μM L-theanine group; *n* ≥ 60 in each group. Group with 2% glucose and group with 2% glucose plus 10 μM L-theanine; *n* ≥ 60 in each group. All groups were tested with N2 strains. (**B**–**D**) show effects on body bending, head thrashing, pharyngeal pumping for Ctrl, L 10 μM, 2% glucose, and 2% glucose + L10 μM groups. Each test involved 10 nematodes, with results replicated more than three times to ensure reliability. Statistical significance between experimental groups was determined using *t*-tests, with a *p*-value of <0.05 considered significant.

This study first evaluated the effects of different concentrations of L-theanine on the lifespan of *C. elegans*. In preliminary experiments, we found that L-theanine at a concentration of 10 μM significantly extended the mean lifespan of *C. elegans* (Supplementary Figure S1 and Table S1). Compared to the untreated group, the lifespan of the L-theanine-treated group increased by approximately 20.01%, indicating a significant anti-aging effect of L-theanine. These replicate experiments showed consistent and significant lifespan extension effects, indicating the reliability and reproducibility of this finding. Furthermore, to investigate the anti-aging effect of L-theanine under high-glucose conditions, we added 2% glucose to the early adult stage of *C. elegans* to induce a high-glucose stress response. Under these high-glucose conditions, the lifespan of *C. elegans* was significantly shortened. However, when 10 μM L-theanine was added to the high-glucose treatment, the lifespan of *C. elegans* was significantly extended. Compared to the group treated with only high glucose, the lifespan of the L-theanine-treated group increased by approximately 15.31%, indicating that L-theanine can alleviate the lifespan-shortening effect of high-glucose conditions (Figure 1A and Table S2).

Phenotypic tests showed reduced body bending in the 2% glucose group compared to the control, while head thrashing and pharyngeal pumping remained unchanged. However, supplementing the 2% glucose group with 10 μM L-theanine led to improvements in all three behavioral phenotypes (Figure 1B,C,D and Table S3).

### 3.2. L-Theanine Reduces Accumulation of AGEs Under High-Glucose Conditions

**Figure 2:**
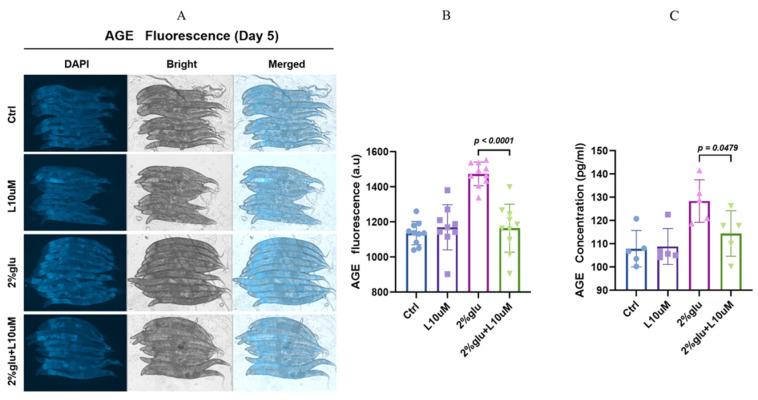
L-theanine reduces AGE accumulation in *C. elegans* under high-glucose conditions. (**A**) Fluorescence imaging at day 5, showing the DAPI, bright, and merged fluorescence levels across the four groups, with each group having *n* = 10. (**B**) Quantitative analysis between groups, analyzed using *t*-tests with a *p*-value < 0.05 indicating significant differences. (**C**) ELISA measurements used to quantify the AGE levels across the different treatment groups in *C. elegans*. Each bar represents the mean AGE concentration, with the error bars indicating the standard deviation; the statistical significance was determined by a *t*-test.

To further investigate the anti-aging effects of L-theanine under high-glucose conditions, we measured the AGE level in *C. elegans*. In this experiment, we set up four treatment groups: (1) A control group under normal culture conditions; (2) a group treated with a 10 μM concentration of L-theanine; (3) a group treated with 2% glucose; and (4) a group treated with 2% glucose and a 10 μM concentration of L-theanine. The nematodes were subjected to these treatments during the early adult stage and analyzed on day 5. The DAPI fluorescence levels observed under a fluorescence microscope reflected the AGE level (Figure 2A). Additionally, we confirmed the quantitative levels of AGEs in the nematodes through a fluorescence quantification analysis. The results showed no significant changes in the control group or the 10 μM L-theanine group under normal conditions. Under the high-glucose treatment, the fluorescence levels of AGEs significantly increased. However, when a 10 μM concentration of L-theanine was added, the fluorescence levels of AGEs decreased by about 26.5%, indicating that L-theanine effectively mitigated the accumulation of AGEs (Figure 2B and Table S4). Further confirmation of the AGE levels in *C. elegans* was obtained through ELISA experiments, which substantiated that a 10 µM concentration of L-theanine can reduce the accumulation of AGEs (Figure 2C and Table S5).

### 3.3. L-Theanine Alleviates Oxidative Stress Under High-Glucose Conditions by Regulating gst-4 Expression

To further verify the anti-aging and detoxifying effects of L-theanine under high-glucose conditions, we measured the fluorescence expression levels of the *gst-4* gene in *C. elegans*. Previous studies have reported that *gst-4* is a key oxidative stress gene, and its expression level can serve as an indicator of oxidative stress.

**Figure 3:**
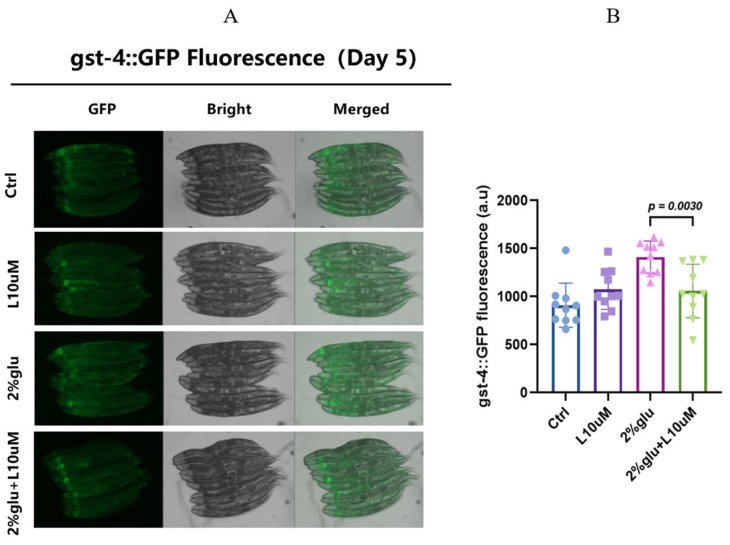
L-theanine reduces the *gst-4* expression in *C. elegans* under high-glucose conditions. (**A**) Fluorescence imaging was performed on day 5, showing the GFP, bright, and merged fluorescence levels across the three groups, with each group consisting of *n* = 10 worms. (**B**) A quantitative analysis was conducted to compare the groups, with the statistical significance determined using a *t*-test, and a *p*-value of <0.05 indicating significant differences.

In this experiment, the worms underwent treatments during the early adult stage, as described in Section 2.2. First, we observed and quantitatively analyzed the expression levels of the *gst-4* gene using fluorescence microscopy at day 5. The results showed that, in the high-glucose environment (2% glucose treatment group), the fluorescence expression of *gst-4* in the worms significantly increased, indicating a substantial oxidative stress response induced by the high-glucose treatment. However, when 10 μM L-theanine was added to the high-glucose treatment, the fluorescence expression of *gst-4* significantly decreased. Compared to the high-glucose treatment group alone, the *gst-4* expression in the L-theanine treatment group decreased by approximately 40% (Figure 3A,B and Table S6). These results suggest that L-theanine can alleviate oxidative stress under high-glucose conditions by reducing the expression of the *gst-4* gene, further confirming its anti-aging and detoxifying effects.

### 3.4. L-Theanine Modulates Lifespan and AGE Accumulation in C. elegans via the daf-2/daf-16 Pathway

**Figure 4:**
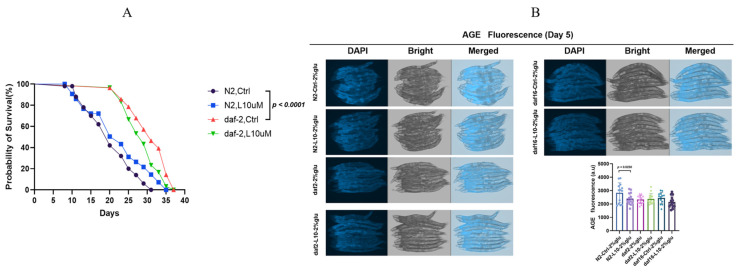
L-theanine regulates the lifespan and AGE accumulation via the *daf-2*/*daf-16* pathway. (**A**) Control group (N2), 10 μM L-theanine group (N2), *daf-2* mutant group, and *daf-2* mutant with 10 μM L-theanine group, with each group consisting of *n* = 60. (**B**) A quantitative analysis between the different groups was performed using a *t*-test, with a *p*-value of <0.05 indicating significant differences. For the fluorescence analysis, 8–10 representative *C. elegans* from each group were selected for presentation.

To explore the relationship between L-theanine and the classic longevity pathway involving *daf-2* and *daf-16* in *C. elegans*, we conducted a series of experiments analyzing the effects of different gene mutants on the worm lifespan and AGE accumulation. Under normal conditions, *daf-2* mutants exhibited a significantly extended lifespan. However, when 10 μM L-theanine was added, the lifespan of *daf-2* mutants did not further increase, indicating that the longevity-extending effect of L-theanine is suppressed in the presence of a *daf-2* gene mutation under normal conditions (Figure 4A and Table S7).

A further fluorescence analysis of AGE accumulation revealed that, under high-glucose conditions, the AGE fluorescence levels in *daf-2* mutants were not significantly reduced compared to the L-theanine-treated group, further validating the weakened effect of L-theanine in regulating the *daf-2* gene (Figure 4B and Table S8). These results suggest that the *daf-2* gene may be a potential key gene for L-theanine’s lifespan-extending and AGE-reducing effects. L-theanine’s role in modulating the *daf-2*/*daf-16* pathway further validates its anti-aging mechanism.

## 4. Discussion

### 4.1. Mechanisms of AGEs and RAGE

This study investigated the potential anti-aging effects of L-theanine in *C. elegans* under high-glucose conditions, focusing on its mechanisms in regulating the lifespan and the accumulation of advanced glycation end products (AGEs). Our research provides new insights into how L-theanine extends the lifespan and reduces AGE accumulation through various pathways, enhancing our understanding of its anti-aging effects.

The insulin/IGF-1 signaling pathway plays a crucial role in regulating growth, metabolism, and aging. In *C. elegans*, the *daf-2* gene encodes a receptor that is a major component of this pathway [40]. Early studies have shown that *daf-2* mutants exhibit significantly extended lifespans due to the inhibition of the IGF-1R signaling pathway, which reduces the transmission of PI3K/AKT signals [41,42]. This inhibition decreases the phosphorylation and activation of downstream effectors, thereby extending the lifespan of *C. elegans* [43,44,45]. The inactivation of *daf-2* also leads to the activation of *daf-16* (FOXO transcription factor), which plays a crucial role in stress responses and lifespan regulation [46,47,48]. Activated *daf-16* enhances an organism’s anti-aging capacity by regulating a series of genes, including those encoding antioxidant enzymes and heat shock proteins [49,50,51]. In our study, we observed that, while L-theanine supplementation did not significantly extend the lifespan of *daf-2* mutants under normal conditions, it significantly shortened their lifespan under high-glucose conditions. This suggests that *daf-2* may have a complex regulatory role in mediating L-theanine’s anti-aging effects. Specifically, under stress-inducing high-glucose conditions, the protective effect of L-theanine on *daf-2* mutants was diminished. Compared to *daf-2* mutants exposed to high glucose alone, those supplemented with 10 μM L-theanine showed a further reduced lifespan. This indicates that the absence of *daf-2* may critically impair L-theanine’s ability to mitigate aging effects under high-glucose conditions, highlighting *daf-2*’s importance in mediating L-theanine’s anti-aging properties under stress.

AGEs are aging markers associated with oxidative damage and glycation, contributing to the progression of age-related diseases by cross-linking proteins and impairing cellular functions [52]. AGEs bind to their receptor, RAGE (receptor for advanced glycation end products), activating intracellular signaling pathways such as NF-κB, MAPK, and JAK/STAT, which induce oxidative stress and inflammatory responses, further promoting tissue damage and disease progression [53,54]. NF-κB, a crucial transcription factor in the RAGE signaling pathway, regulates genes involved in inflammation and immune responses [55,56]. Upon AGEs binding to RAGE, NF-κB is activated and translocated to the nucleus, inducing the expression of inflammatory factors like TNF-α, IL-6, and ICAM-1. The overproduction of these inflammatory factors exacerbates oxidative stress, promotes apoptosis, and causes tissue damage, leading to age-related diseases [57,58].

Additionally, AGE–RAGE interactions activate the MAPK and JAK/STAT signaling pathways, enhancing inflammation and cellular stress [59,60]. Our study, using fluorescence microscopy and a quantitative analysis, found that L-theanine significantly reduced the AGE fluorescence levels in *C. elegans* under high-glucose conditions (Figure 2B). This finding aligns with other studies suggesting that dietary or pharmacological interventions targeting AGEs can mitigate aging-related phenotypes. L-theanine likely exerts its anti-aging effects by inhibiting AGE formation, reducing their binding to RAGE, and disrupting RAGE-mediated signaling pathways. Besides L-theanine, several other compounds have been found to reduce AGE accumulation. For example, aminoguanidine is an effective AGE inhibitor that reacts with AGE precursors, preventing AGE formation [61,62]. Natural antioxidants like quercetin and resveratrol have also been shown to reduce AGE production and alleviate AGE-induced cellular damage through their antioxidant and anti-inflammatory properties [63,64].

### 4.2. Mechanisms of gst-4 Gene Expression in Reducing Oxidative Stress

This study also investigated the mechanism by which L-theanine modulates *gst-4* gene expression to reduce oxidative stress under high-glucose conditions. GST-4 is a glutathione S-transferase that plays a critical role in the oxidative stress response [65]. Oxidative stress refers to an imbalance between the production and clearance of reactive oxygen species (ROS), leading to cellular damage. ROS, including superoxide, hydrogen peroxide, and hydroxyl radicals, can cause damage to proteins, lipids, and DNA. Oxidative stress is closely associated with various age-related diseases, such as cardiovascular diseases, neurodegenerative diseases, and cancer [66].

Reducing ROS levels and enhancing the antioxidant capacity are considered important strategies for delaying aging. In addition to L-theanine, other compounds such as N-acetylcysteine (NAC), vitamin C, and vitamin E have demonstrated significant antioxidant effects [67,68]. NAC, an antioxidant precursor, enhances the cellular antioxidant capacity by providing cysteine for glutathione synthesis [69]. Glutathione is an endogenous antioxidant that plays a crucial role in ROS scavenging, maintaining the cellular redox environment, and repairing oxidative damage.

Vitamin C and vitamin E directly scavenge free radicals, protecting cells from oxidative damage [70]. Vitamin C (ascorbic acid) is a water-soluble antioxidant that reduces ROS by donating electrons, preventing the oxidation of DNA, proteins, and lipids [71]. Vitamin E (α-tocopherol) is a fat-soluble antioxidant that embeds in cell membranes, protecting membrane lipids from oxidative damage [72]. In our experiments, the *gst-4* expression significantly increased in *C. elegans* under high-glucose conditions, indicating a pronounced oxidative stress response. However, upon L-theanine supplementation, the *gst-4* expression significantly decreased. Compared to the high-glucose treatment group, the L-theanine-treated group showed about a 40% reduction in *gst-4* expression (Figure 3). This suggests that L-theanine may alleviate oxidative stress by reducing *gst-4* expression, thereby decreasing ROS production.

## 5. Conclusions

Our study provides novel insights into the mechanisms underlying L-theanine’s anti-aging effects in *C. elegans*. By targeting pathways such as *daf-2*/*daf-16* and modulating oxidative stress through *gst-4*, L-theanine extends the lifespan and reduces AGE accumulation under stress conditions. This type of study faces several challenges. Investigating the interplay between L-theanine and *daf-2* signaling to clarify their mechanistic relationship in aging is complex due to the intricate nature of aging processes. Moreover, exploring the translational potential of L-theanine in mammalian models is challenging, as it requires accurate model selection, the consideration of species differences, and ensuring that the findings can be effectively applied to develop therapies for age-related diseases.

## Data Availability

The original contributions presented in this study are included in the article/Supplementary Materials. Further inquiries can be directed to the corresponding authors.

## Supplementary Materials

The following supporting information can be downloaded at: https://www.mdpi.com/article/10.3390/foods14020221/s1, Figure S1: Lifespan curve of L-theanine at different concentrations for *C. elegans*; Table S1: Lifespan curve of L-theanine at different concentrations for *C. elegans*; Table S2: Effects of L-Theanine on Lifespan phenotypes of *C. elegans*; Table S3: Effects of L-Theanine on phenotypes of *C. elegans*; Table S4: L-Theanine Reduces Lipofuscin/AGEs Accumulation in *C. elegans* Under High Glucose Conditions; Table S5: ELISA Analysis of AGE Concentrations in *C. elegans*; Table S6: L-Theanine Reduces gst-4 Expression in *C. elegans* Under High Glucose Conditions; Table S7: L-Theanine Regulates Lifespan via the DAF-2/DAF-16 Pathway; Table S8: L-Theanine Regulates AGEs Accumulation via the DAF-2/DAF-16 Pathway.
